# Evaluating an implementation strategy in cardiovascular prevention to improve prescribing of statins in Germany: an intention to treat analysis

**DOI:** 10.1186/1471-2458-13-623

**Published:** 2013-07-02

**Authors:** Heidemarie Keller, Oliver Hirsch, Petra Kaufmann-Kolle, Tanja Krones, Annette Becker, Andreas C Sönnichsen, Erika Baum, Norbert Donner-Banzhoff

**Affiliations:** 1 Department of General Practice/Family Medicine, Philipps University of Marburg, Karl-von-Frisch-Strasse 4, Marburg, 35043, Germany; 2 AQUA-Institute for Applied Quality Improvement and Research in Health Care, Göttingen, Germany; 3 Clinical Ethics, University Hospital Zurich & Institute of Biomedical Ethics, University of Zurich, Zurich, Switzerland; 4 Institute of General Practice, Family Medicine and Prevention, Paracelsus Medical University, Salzburg, Austria

**Keywords:** Evaluation studies, Intention to treat analysis, Cardiovascular diseases, Drug prescriptions, Hydroxymethylglutaryl-CoA Reductase Inhibitors

## Abstract

**Background:**

The prescription of statins is an evidence-based treatment to reduce the risk of cardiovascular events in patients with elevated cardiovascular risk or with a cardiovascular disorder (CVD). In spite of this, many of these patients do not receive statins.

**Methods:**

We evaluated the impact of a brief educational intervention in cardiovascular prevention in primary care physicians’ prescribing behaviour regarding statins beyond their participation in a randomised controlled trial (RCT). For this, prescribing data of all patients > 35 years who were counselled before and after the study period were analysed (each n > 75000). Outcome measure was prescription of Hydroxymethylglutaryl-CoA Reductase Inhibitors (statins) corresponding to patients’ overall risk for CVD. Appropriateness of prescribing was examined according to different risk groups based on the Anatomical Therapeutic Chemical Classification System (ATC codes).

**Results:**

There was no consistent association between group allocation and statin prescription controlling for risk status in each risk group before and after study participation. However, we found a change to more significant drug configurations predicting the prescription of statins in the intervention group, which can be regarded as a small intervention effect.

**Conclusion:**

Our results suggest that an active implementation of a brief evidence-based educational intervention does not lead to prescription modifications in everyday practice. Physician’s prescribing behaviour is affected by an established health care system, which is not easy to change.

**Trial registration:**

ISRCTN71348772

## Background

The prescription of statins is an evidence-based treatment to reduce the risk of cardiovascular events in patients with elevated cardiovascular risk or with a cardiovascular disorder [1-4]. Nevertheless, many of those patients do not receive statin therapy [5-8]. In a German study, Berthold et al. found that the majority of patients with type 2 diabetes were not receiving statins. They were more likely to be prescribed a statin in secondary prevention. In primary prevention, this depended on the individual cardiovascular risk status. The authors conclude that physicians were generally aware of the concept of cardiovascular risk, but they did not consistently implement evidence-based treatments [9]. In an Italian sample less than 40% of eligible patients were prescribed statins and less than half of those receiving statins were taking wrong doses [10]. There is also a low adherence to statin therapy in patients [10,11], which highlights the importance of providing adequate information.

According to existing guidelines in the prevention of cardiovascular disease (CVD) [12-14], we developed arriba^TM^, a simple, Framingham-based [15] educational intervention that combines risk calculation and consultation based on a patient’s individual global risk for CVD to help practitioners accommodate for the double paradigm shift towards global cardiovascular risk and shared decision making (SDM) [16]. SDM incorporates evidence-based medicine and was shown to increase adherence to medical treatment decisions [17].

We had conducted a pragmatic cluster randomized controlled trial (RCT) and had chosen an active implementation strategy addressing continuing medical education (CME) groups [18]. Prior to the start of the trial, arriba™ had undergone a three year phase I/II development process [19]. We had discussed the epidemiological background of global CVD risk calculation and the ethics of shared decision making (SDM) with an emphasis on practical communication strategies and materials to be applied during consultation. Results on main outcome measures have been recently published, including details on power calculations, recruitment rates, and baseline characteristics [16,20,21]. An intervention effect on prescribing could only be found for inhibitors of platelet aggregation, independent from individual cardiovascular risk. No changes were observed for statin prescription rates [21]. The active implementation of a brief, evidence-based educational intervention on the global risk of CVD did not directly lead to risk-adjusted changes in prescription within a period of 6 months. Therefore, the intention was to increase the time frame regarding statin prescription behavior and to examine whether decision support of this kind would improve the risk-adjusted prescription of this medication.

For assessing appropriateness of prescription, it is important to correctly identify the respective target population, i.e. patients with a specific condition who should receive a specific treatment. It has been recognized that the data source used can influence the outcome of the quality assessment. Often either diagnostic codes or clinical measurements are used to identify target patients [22]. The selective recruitment of patients for RCTs might contain such problems.

In this article we introduce an approach to evaluate our implementation strategy on the appropriateness of prescription beyond study participation in our RCT and for a longer time frame than our previous study. For this, we included prescribing data of all patients > 35 years who had been counselled before and after the study period. We used this data to investigate the effectiveness of the intervention by applying an intention to treat analysis [23] approach.

## Methods

### Study sample

The majority of German family doctors are organised in continuing medical education (CME) groups. Hessian CME groups supervised by AQUA-institute, a German quality management institute, had been offered to participate in the initial RCT (http://www.aqua-institut.de). We refer to the original physician sample of our RCT [16]. We excluded CME groups if several members had already taken part in previous meetings on arriba^TM^, or if they had routinely used other cardiovascular risk calculators at that time. As a result, we attained 14 CME groups with 162 physicians. Randomisation of the physicians to intervention or control group was performed on CME group level by the Centre for Clinical Trials, University of Marburg, Germany. Participating general practitioners (GPs) of the intervention arm were invited to attend two CME sessions lasting 2 1/2 hours each. The epidemiological background of global cardiovascular risk calculation, as well as the ethics of shared decision making, were discussed by emphasising practical communication strategies and materials (arriba^TM^) to be applied during consultation. Concurrently, GPs of the control arm were invited to participate in seminars on defined alternative topics not related to cardiovascular prevention. More details about the intervention can be found elsewhere [20].

After completing the educational sessions, participating physicians were asked to recruit a maximum of 15 of their adult patients for the RCT. Rolling recruitment of patients was spread evenly from May 2005 to March 2006.

**Figure 1:**
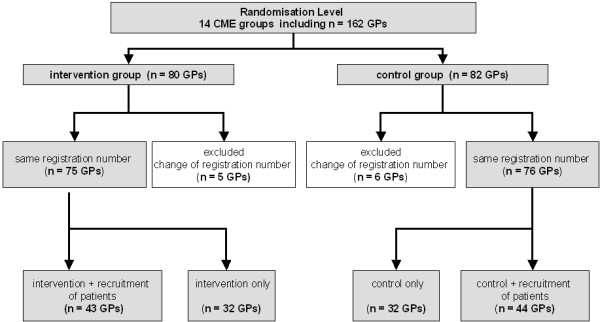
Flow chart of participating GPs.

For the present evaluation, all GPs who participated in our RCT and who were registered in the Hessian Association of Statutory Health Insurance Physicians before study start (baseline = 1st quarter 2005) as well as after study end (evaluation = 1st quarter 2006) and had received continuing education were integrated in the present investigation (Figure 1), independent of patient recruitment in our RCT. Of those, 11 GPs changed their registration number during the examination period, which made data analysis impossible for those cases (intervention group n = 5, control group n = 6). This resulted in a total of 75 GPs in the intervention arm (43 GPs recruited patients, 32 GPs did not) and 76 GPs in the control arm (44 GPs recruited patients, 32 did not). Independent of whether a GP had recruited study patients, all GPs were considered for this analysis because of the ITT approach. Figure 1 shows characteristics of the physician sample.

The study complies with the Declaration of Helsinki. The research protocol was approved by the local research ethics committee, University of Marburg, Germany. Informed consent was obtained from all participating GPs.

### Measures

We evaluated changes in prescribing behaviour using ITT analysis since the only available data source for evaluation was prescribing data from each participating GP between January 2005 and December 2006; the data was not linked to patients’ diagnoses. Data retrieval was possible through the billing of Statutory Health Insurance-accredited physician services with regard to the German Social Security Statute Book (§ 295 SGB V). Prescription was examined according to different cardiovascular risk groups based on the Anatomical Therapeutic Chemical Classification System (ATC codes) [24] as displayed in Table 1. We defined four cardiovacular risk groups (very high, high, intermediate, low) based on the prescription of drugs with specific ATC codes. We validated this algorithm on the basis of data of our RCT [16] with the arriba^TM^ risk calculation. The average cardiovascular risk in the very high risk group was 35%, in the high risk group it was 29%, in the intermediate risk group it was 18%, and in the low risk group it was 7%. Outcome measure was prescription of Hydroxymethylglutaryl-CoA Reductase Inhibitors (statins - ATC code C10AA). Prescription of statins was considered to be appropriate in patients of risk groups 1 (very high risk) and 2 (high risk), non-prescription of statins was considered to be appropriate in patients of risk groups 3 (intermediate risk) and 4 (low risk).

**Table 1:** Risk group classification for cardiovascular disease (CVD) according to the anatomical therapeutic chemical classification system (ATC codes)

**Risk group 1 = patients with very high risk**	
**ATC Code**	**Agents**
C01DA	Vasodilators used in cardiac diseases (organic nitrates)
	*or*
B01AC04	Clopidogrel
	*or*
B01AC05	Ticlopidine
	*or*
C02 and/or C03 and/or C07 and/or C08 and/or C09	Antihypertensives, diuretics, beta blocking agents, calcium channel blockers, agents acting on the renin-angiotensin system
	*and*
A10	Antidiabetics
	*and*
	Male > 49 years or female > 54 years
**Risk group 2 = patients with high risk**	
**ATC Code**	**Agents**
C02	Antihypertensives
	*or*
C03	Diuretics
	*or*
C07	Beta blocking agents
	*or*
C08	Calcium channel blocker
	*or*
C09	Agents acting on the renin-angiotensin system
	*or*
A10	Antidiabetics
	*or*
B01AA	Vitamin K antagonists
	*or*
B01AC06	Acetyl salicylic acid
	*and*
	Male > 49 years or female > 54 years
**Risk group 3 = patients with intermediate risk**	
**ATC Code**	**Agents**
C02	Antihypertensives
	*or*
C03	Diuretics
	*or*
C07	Beta blockers
	*or*
C08	Calcium channel blocker
	*or*
C09	Agents acting on the renin-angiotensin system
	*or*
A10	Antidiabetics
	*or*
B01AA	Vitamin K antagonists
	*or*
B01AC06	Acetyl salicylic acid
**Low risk group 4 = all the others**	
**ATC Code**	**Agents**
	All patients with medication > 35 years

### Statistical analysis

We calculated Cochrane-Mantel-Haenszel tests in order to examine the association between group allocation of physicians and statin prescription controlling for risk status of patients [23]. After stratifying on the observed covariates, the Cochrane-Mantel-Haenszel (CMH) test is used to measure the strength of the association between an exposure and a disease or response. The rows of the resulting table correspond to the treatment group and the columns to the dependent values. The alternative hypothesis is that the statin prescription is conditionally dependent of the treatment in any given strata. If this is the case, the direction of such a significant difference must be examined. The resulting effect is evaluated by the effect size Cramer V. A Cramer V of .40 or higher denotes a large effect [25,26].

Within the high risk group, we performed a prediction configural frequency analysis (CFA) with Bonferroni-Holm correction [27] to identify drug configurations which predicted the description or non-description of statins [28,29]. The non-parametric configural frequency analysis is a multivariate statistical method used to explore associations among categorical variables. With this approach it is possible to categorize subjects and reveal reciprocal dependencies between certain features or characteristics. Furthermore, it is possible to examine the influence of independent variables on dependent variables (prediction Configural Frequency Analysis). It can also be used to generate hypotheses (explorative CFA) and for hypothesis testing (confirmatory CFA). Analyses were performed using SPSS 17.0 and CFA [29].

## Results

### Prescribing patterns

We performed the Cochrane-Mantel-Haenszel test in order to examine the association between group allocation and statin prescription controlling for risk status before the start of our RCT. The Breslow-Day test indicates the homogeneity of the odds ratios (χ^2^ =3.91, p = .27). The Cochrane-Mantel-Haenszel test signals a significant association between group allocation and statin prescription after controlling for risk status (χ^2^ =15.05, p < .001). Comparing the intervention with the control group, a common odds ratio of .894 (95%CI: .845 - .946) means that the chance of a statin prescription is reduced by 10.6% (95%CI: 5.4% - 15.5%) in the intervention group. The distributions in the intervention and control group are very similar within the different risk groups. The significant result most probably emerged because of the known sample size dependence of χ^2^. The raw data before the intervention with arriba^TM^ are presented in Table 2.

**Table 2:** **Cross-tabulation of risk status, group allocation, and statin prescription *before *the intervention with arriba**^**TM**^

**Risk group**			**Statins**		**Total**	**Odds ratios**
			**No**	**Yes**		**(95% confidence interval)**
Group 1very high risk	Intervention	n	3745	958	4703	
		%	79.6%	20.4%	100.0%	
	Controls	n	3534	878	4412	0.97
		%	80.1%	19.9%	100.0%	(0.88-1.08)
Group 2 high risk	Intervention	n	12929	1433	14362	
		%	90.0%	10.0%	100.0%	
	Controls	n	11647	1125	12772	0.87
		%	91.2%	8.8%	100.0%	(0.80-0.95)
Group 3 intermediate risk	Intervention	n	2496	132	2628	
		%	95.0%	5.0%	100.0%	
	Controls	n	2412	104	2516	0.82
		%	95.9%	4.1%	100.0%	(0.63-1.06)
Group 4 low risk	Intervention	n	16717	469	17186	
		%	97.3%	2.7%	100.0%	
	Controls	n	16417	391	16808	0.85
		%	97.7%	2.3%	100.0%	(0.74-0.97)

We again performed the Cochrane-Mantel-Haenszel test in order to examine the association between group allocation and statin prescription controlling for risk status after the intervention. The raw data after the intervention with arriba^TM^ are presented in Table 3.

**Table 3:** **Cross-tabulation of risk status, group allocation, and statin prescription *after *the intervention with arriba**^**TM**^

**Risk group**			**Statins**		**Total**	**Odds ratios**
			**No**	**Yes**		**(95% confidence interval)**
Group 1 very high risk	Intervention	n	3905	1183	5088	
		%	76.7%	23.3%	100.0%	
	Controls	n	3409	1069	4478	1.04
		%	76.1%	23.9%	100.0%	(0.94-1.14)
Group 2 high risk	Intervention	n	14003	1735	15738	
		%	89.0%	11.0%	100.0%	
	Controls	n	12383	1469	13852	0.96
		%	89.4%	10.6%	100.0%	(0.89-1.03)
Group 3 intermediate risk	Intervention	n	2686	149	2835	
		%	94.7%	5.3%	100.0%	
	Controls	n	2459	127	2586	0.93
		%	95.1%	4.9%	100.0%	(0.73-1.19)
Group 4 low risk	Intervention	n	16823	538	17361	
		%	96.9%	3.1%	100.0%	
	Controls	n	15724	477	16201	0.95
		%	97.1%	2.9%	100.0%	(0.84-1.08)

The Breslow-Day test indicates the homogeneity of the odds ratios (χ^2^ =2.09, p = .55). The Cochrane-Mantel-Haenszel test reveals no significant association between group allocation and statin prescription after controlling for risk status (χ^2^ =0.76, p = .38). There was still a low supply with statins in the very high risk intervention group (23.3%), especially in the high risk intervention group (11.0%). Therefore, we used a prediction configural frequency analysis to investigate which configuration predicted the prescription or the non-prescription of statins in the high risk group before the start of our RCT. The significant prediction configurations are displayed in Table 4.

**Table 4:** **Significant types in a prediction configural frequency analysis (P-CFA) *before *the intervention with arriba**^**TM**^**; DV = dependent variable, p = level of significance**

**Antidiabetics**	**Ticlopidine**	**Clopidogrel**	**Nitrates**	**Statins DV**	**p intervention group**	**p control group**
No	No	No	Yes	No	p = .0002	------
No	No	Yes	No	No	p < .000001	------
No	No	Yes	No	Yes	------	p < .000001
No	No	Yes	Yes	Yes	p < .000001	p = .0005
Yes	No	No	No	No	p = .000004	------
Yes	No	No	Yes	No	p = .0005	------
Yes	No	Yes	No	Yes	------	p < .000001

The two groups differ in their prediction configurations in that the prescription of statins in the control group is predicted by three configuration types consisting of patients with clopidogrel, with clopidogrel and nitrate, and with antidiabetics and clopidogrel. In contrast, we mainly found configurations that prevented the physicians from prescribing statins in the intervention group. These are patients with nitrates, with clopidogrel, with antidiabetics, and with antidiabetics and nitrates.

Using a prediction configural frequency analysis, we again investigated which configuration predicted the prescription or the non-prescription of statins in the high risk group after the intervention with arriba^TM^. The significant prediction types are displayed in Table 5.

**Table 5:** **Significant configurations in a prediction configural frequency analysis (P-CFA) *after *the intervention with arriba**^**TM**^**; DV = dependent variable, p = level of significance**

**Antidiabetics**	**Ticlopidine**	**Clopidogrel**	**Nitrates**	**Statins DV**	**p intervention group**	**p control group**
No	No	No	Yes	No	p = .00009	p = .0014
No	No	Yes	No	Yes	p < .000001	p < .000001
No	No	Yes	Yes	Yes	------	p = .0006
Yes	No	No	No	No	p < .000001	p < .000001
Yes	No	No	Yes	Yes	p < .000001	------
Yes	No	Yes	No	Yes	p < .000001	p < .000001
Yes	No	Yes	Yes	Yes	------	p = .0003

The distribution of significant prediction types changed in both groups. In the intervention group there was a change to significant configurations predicting the prescription of statins: patients with clopidogrel, with antidiabetics and nitrates, and with antidiabetics and clopidogrel.

In both groups the sole prescription of nitrates or of antidiabetics predicted the non-prescription of statins. Generally, the number of positive prediction configurations (statin - yes) has increased in the intervention arm, and the number of negative prediction configurations (statin - no) has increased in the control group. Regarding the change in the intervention group, this might be classified as an effect of the intervention. Nevertheless, both groups are quite similar in their medication patterns at the end of the observed time period.

### Appropriateness of prescription

To examine the general appropriateness of statin prescription we grouped patients with very high risk and high risk receiving statins, and those with intermediate and low risk not receiving statins in intervention and control physicians. These prescriptions were labelled as “appropriate”. We then grouped patients with very high risk and high risk not receiving statins, and those with intermediate and low risk receiving statins in intervention and control physicians. These prescriptions were labelled as “not appropriate”.

The χ^2^ –test signals a significant association between group and appropriateness of prescription (χ^2^ (df = 1) = 17.09, p < .001) before the start of our RCT. This significant result might be due to the sample size dependence of χ^2^ as the corresponding effect size Cramer V with .015 denotes a negligible effect. The odds ratio of 0.94 (CI 95%: 0.92-0.97) indicates a slightly more appropriate statin prescription in the control group.

The χ^2^ test still shows a significant association between group and appropriateness of prescription (χ^2^ (df = 1) = 10.52, p = .001) after the intervention. This can again be interpreted in the context of the sample size dependence of χ^2^ as the corresponding effect size Cramer V with .012 indicates a negligible effect. The odds ratio of 0.95 (CI 95%: 0.93-0.98) again points to a more appropriate prescription in the control group.

## Discussion

### Effects on prescription

The educational intervention of our underlying RCT aimed for a double paradigm shift towards shared decision making and rational global cardiovascular risk management. It had a significant impact on the communication behaviour of participating GPs as recently published [16], but there was hardly any effect on prescription behaviour [21]. In order to evaluate our intervention regarding our entire physician sample, regardless of patient recruitment for our RCT, we analyzed prescribing data using prescription of statin as a measurement index.

Main findings before our RCT were independent of group allocation: the higher the risk category, the more statins were prescribed, but much less than expected and required by guidelines. The distribution of appropriate risk-adapted medication (e.g., very high or high risk: statin; intermediate or low risk: no statin) in the intervention and control group are very similar among the different risk groups. The observed low prescribing rates of statins in high and very high risk patients were also independent of group allocation. On the one hand, there might have been a misclassification of risk due to inadequate prescribing of nitrates without definite diagnosis of CVD. In addition, vitamin K antagonists might have been prescribed for venous thromboembolism compatible with low CVD risk, as well as diuretics for peripheral oedema without hypertension or heart failure. On the other hand, there might have been a misclassification regarding our outcome parameter. Statins prescribed by other physicians or in another quarter of the year than we analyzed would not necessarily have been captured by our study.

We did not explore reasons for lacking guideline adherence. A high risk patient not on statin medication may have had side effects in the past, discontinued medication on his own, refused to take medication, or preferred to try behavioural changes first. A quality problem might have occurred because of a prescription not based on global cardiovascular risk. Consequently, it might also have been possible that a patient with a low cholesterol level was not prescribed a statin although his global CVD risk was high.

However, the change to more significant configurations predicting the prescription of statin in the intervention group can be regarded as a small intervention effect. The physicians in both groups were not aware that the configurations were monitored, so that decisions in the expected direction could not be evoked artificially. However, using different outcome measurements, our findings here still demonstrate a gap between recommended and current drug use for primary prevention of CVD according to global risk as reported by Wensing et al. [18].

### Interpretation of results

A recent study has shown that measurement indices like those applied in our study are even better when used as a prescribing quality indicator (PQI) in comparison to parameters based on diagnoses [22]. Although we provided intervention GPs with information on how to treat diseases in accordance with research evidence and guideline recommendations, this was insufficient to change prescribing behaviour. In a recently published primary care physician survey, the authors found that providing 10 year coronary risk information improved some hypothetical prescribing of acetylsalicylic acid and also improved lipid management when the CVD risk was moderately high [30]. Others identified acetylsalicylic acid underuse by some patients with increased risk and potentially inappropriate use by some with low risk [31-33]. Such findings align with our results and suggest that specific guideline recommendations should be provided along with clinical decision support and risk assessment. Therefore, our intervention period might have been too short to initiate such changes. Our findings are supported by a comparison to prescribing data of a total of 1 519 722 patients derived from the Hessian Association of Statutory Health Insurance Physicians, including all other GPs who had also been registered in both investigation periods. Using the same methodological approach, we found a matchable low supply of statins in the very high (22.6%) and high risk (11.2%) groups. Likewise, patients in groups with intermediate (95.5% with no statin prescription) and low cardiovascular risk (97.3% with no statin prescription) were treated similarly to the patients of our participating GPs.

It is well known that successful implementation of innovative behaviour among professionals seems to be more likely contingent upon various external factors such as patients’ expectations, pressure from the wider medical environment, or marketing by drug companies [30,34,35]. Indeed, change requires long-running comprehensive approaches at different levels of the health care system, tailored to specific settings and target groups [36,37]. Despite this, and even with some improvement in knowledge, patient acceptance of and adherence to treatment recommendations may remain suboptimal as inaccurate perceptions of vulnerability to a disease can inhibit behavioural changes [38-40].

The remarkably low prescribing rates of statins in high and very high risk patients might also imply methodological aspects such as a non-registered prescription at the end of a quarter or prescription by specialists, neither of which could have been identified here.

Important research findings often do not translate automatically into practice. Even a comprehensive implementation programme was not able to raise statin prescription rates in eligible patients [5]. Thus, implementation requires a clear and deliverable evidence-based message, evidence that current care is suboptimal, a robust estimate of the cost and impact of alternative methods of behavioural change, and an understanding of the local organization of health care [41]. More attention has to be paid to the validation of different theories on changing professional and organisational performance (from health promotion, social sciences, organisational and management sciences, marketing, and economy) to find the crucial determinants of effective change [37].

### Strengths and limitations

For reliable assessment of prescribing quality it is important to correctly identify the patients eligible for recommended treatment. Implementation studies usually recruit individual patients after intervention for evaluation purpose. Therefore, they have to face the problem of selection bias and concealment of allocation. In our study, evaluation could be performed independently of patient recruitment and patients’ diagnosis because of the availability of routine prescribing data provided by the Hessian Association of Statutory Health Insurance Physicians. However, since the only available data source was not linked to patients’ diagnoses, data analysis was limited. Risk group classification equal to the RCT was impossible, although we validated our algorithm on data of our RCT. In addition, data analysis per protocol was not feasible. As a new approach, prescribing behaviour was examined according to different cardiovascular risk groups based on the Anatomical Therapeutic Chemical Classification System. Such an approach is admissible in order to discover important aspects indirectly connected to the original study and also may enhance external validity [42]. Additionally, prescription data was shown to result in reliable prevalence estimates, especially in cardiovascular diseases [43].

Our data dates back to 2005 and 2006. Nevertheless, the clinical effectiveness and safety of statins have not changed over the last years, neither has the approach based on absolute cardiovascular risk. Lower target levels are favoured in some countries but these are minor changes that do not generally threaten the relevance of our work.

The knowledge and skills of the GPs regarding statin prescription prior to the study can be assumed to be at a similar level. This is a frequent topic in CME courses and in central journals (http://www.aerzteblatt.de/). We reached a balanced distribution of prior knowledge regarding statins in both groups because of randomisation.

## Conclusions

Our results suggest that an active implementation of a brief, evidence-based educational intervention does not immediately lead to a modification in statin prescription in everyday practice. Our findings indicate this may be the case because physicians are embedded in an organisational framework which prevents them from changing prescription behaviour rapidly. The accordance between GPs’ prescribing behaviour as study participants and prescription in daily routine reveals a stable behaviour, which is not easy to change.

Long-term and subgroup analyses are needed to investigate the effects of educational interventions on prescribing behaviour.

## Competing interest

The authors declare that they have no conflict of interest.

## Authors’ contributions

NDB, EB, AS, AB, and TK developed the study. HK, OH and PKK undertook the study. OH and HK analyzed the data. HK and OH wrote the manuscript with all authors contributing to it. All authors read and approved the final manuscript.

## Pre-publication history

The pre-publication history for this paper can be accessed here:

http://www.biomedcentral.com/1471-2458/13/623/prepub
